# Exploring the Anticancer Potential of *Premna resinosa* (Hochst.) Leaf Surface Extract: Discovering New Diterpenes as Heat Shock Protein 70 (Hsp70) Binding Agents

**DOI:** 10.3390/plants12132421

**Published:** 2023-06-22

**Authors:** Valentina Parisi, Giuliana Donadio, Maria Laura Bellone, Soumia Belaabed, Ammar Bader, Angela Bisio, Valeria Iobbi, Erica Gazzillo, Maria Giovanna Chini, Giuseppe Bifulco, Immacolata Faraone, Antonio Vassallo

**Affiliations:** 1 Department of Pharmacy, University of Salerno, Via Giovanni Paolo II 132, 84084 Fisciano, Italy; vparisi@unisa.it (V.P.); gdonadio@unisa.it (G.D.); mbellone@unisa.it (M.L.B.); egazzillo@unisa.it (E.G.); bifulco@unisa.it (G.B.); 2 Department of Chemistry, Research Unit, Development of Natural Resources, Bioactive Molecules Physicochemical and Biological Analysis, University Brothers Mentouri, Route Ain ElBey, Constantine 25000, Algeria; soumia.belaabed@umc.edu.dz; 3 Department of Pharmacognosy, Faculty of Pharmacy, Umm Al-Qura University, Makkah 21955, Saudi Arabia; ambader@uqu.edu.sa; 4 Department of Pharmacy, University of Genova, Viale Cembrano 4, 16148 Genova, Italy; bisio@difar.unige.it (A.B.); valeria.iobbi@edu.unige.it (V.I.); 5 Department of Biosciences and Territory, University of Molise, C.da Fonte Lappone, 86090 Pesche, Italy; mariagiovanna.chini@unimol.it; 6 Department of Science, University of Basilicata, Viale dell’Ateneo Lucano 10, 85100 Potenza, Italy; immacolata.faraone@unibas.it; 7 Innovative Startup Farmis s.r.l., Via Nicola Vaccaro 40, 85100 Potenza, Italy; 8 Spinoff TNcKILLERS s.r.l., Viale dell’Ateneo Lucano 10, 85100 Potenza, Italy

**Keywords:** *Premna resinosa*, Lamiaceae, labdane diterpenes, DFT/NMR, molecular docking, Hsp70

## Abstract

*Premna*, a genus consisting of approximately 200 species, predominantly thrives in tropical and subtropical areas. Many of these species have been utilized in ethnopharmacology for diverse medicinal applications. In Saudi Arabia, *Premna resinosa* (Hochst.) Schauer (Lamiaceae) grows wildly, and its slightly viscid leaves are attributed to the production of leaf accession. In this study, we aimed to extract the surface accession from fresh leaves using dichloromethane to evaluate the anticancer potential. The plant exudate yielded two previously unknown labdane diterpenes, Premnaresone A and B, in addition to three already described congeners and four known flavonoids. The isolation process was accomplished using a combination of silica gel column chromatography and semi-preparative HPLC, the structures of which were identified by NMR and HRESIMS analyses and a comparison with the literature data of associated compounds. Furthermore, we employed a density functional theory (DFT)/NMR approach to suggest the relative configuration of different compounds. Consequently, we investigated the possibility of developing new chaperone inhibitors by subjecting diterpenes **1**–**5** to a Surface Plasmon Resonance-screening, based on the knowledge that oridonin, a diterpene, interacts with Heat Shock Protein 70 (Hsp70) 1A in cancer cells. Additionally, we studied the anti-proliferative activity of compounds **1**–**5** on human Jurkat (human T-cell lymphoma) and HeLa (epithelial carcinoma) cell lines, where diterpene 3 exhibited activity in Jurkat cell lines after 48 h, with an IC_50_ of 15.21 ± 1.0 µM. Molecular docking and dynamic simulations revealed a robust interaction between compound **3** and Hsp70 key residues.

## 1. Introduction

The *Premna* genus, belonging to the Lamiaceae family, encompasses approximately 200 species that are primarily distributed across tropical and subtropical regions of Asia, Africa, Australia, and the Pacific Islands [1]. In the Flora of China, 46 species have been identified, while the Flora Malesiana recognizes 14 species [1]. These species are predominantly small trees or shrubs, although they can be found as lianas and pyroherbs to a lesser extent [1]. The most common metabolites found within the genus include diterpenoids, triterpenoids, iridoid glycosides, flavonoids, lignans, and xanthones [1,2].

The diverse array of *Premna* species in their natural habitat has led to various traditional uses by local communities [1]. These plants have been employed in treating a range of ailments, including malaria, stomach and hepatic disorders, headaches, coughs, tuberculosis, infectious-related diseases, skin conditions, asthma, rheumatism, neuralgia, diarrhea, hyperglycemia, and obesity [1,2]. Extracts and isolated compounds derived from the leaves, root bark, and stem bark of these plants have exhibited antioxidant, antibacterial, anti-inflammatory, cytotoxic, antifeedant, and hepatoprotective activities [1,2].

Of particular interest is *Premna resinosa* (Hochst.) Schauer (Lamiaceae), a shrub or small bushy tree characterized by whitish stems and coriaceous leaves with a pleasant aroma. This plant holds significant aromatic value in Saudi Arabia, where it is commonly known as “Shaqab”. The fruits are consumed raw and are also a food source for primates and birds [3]. Additionally, the stems are utilized for imparting fragrance to tea, meat, and butter and are burned as frankincense [3]. Researchers have conducted various phytochemical and pharmacological studies on the plant growing in Saudi Arabia, revealing that the methanolic extract of its aerial parts exhibits anticancer, antimicrobial, and antioxidant activities attributed to flavonoids and triterpenes [4]. Furthermore, the methanolic extracts of stems and leaves have demonstrated anti-angiogenic and cytotoxic effects in rat aortic ring and MTT assays, respectively [5]. The anti-angiogenic activity has been further confirmed through in vivo models, namely, zebrafish embryos and chick chorioallantoic membrane assays, with this activity being attributed to iridoid glycosides [6].

In recent years, the flora of Saudi Arabia has garnered attention from researchers investigating the biological properties of medicinal plants. These studies have revealed that Saudi plants are rich in bioactive secondary metabolites possessing anticancer activity [7,8,9,10,11].

The objective of this study is to isolate specialized metabolites from the fresh leaf exudate of *P. resinosa*, as several studies have highlighted the potential anticancer properties of plant exudates [12,13,14,15,16]. Previous research has focused on investigating the polar fractions of this species, elucidating their anti-angiogenic effect through the presence of iridoid glycosides such as bundlejoside A5 and saccatoside [6]. 

In the present study, we explore the less polar metabolites extracted from the leaf surface. In the literature, numerous plants with anticancer activity have been reported to possess multiple mechanisms of action due to the diversity of phytoconstituents in different organs of the same plants [16,17]. It is worth noting that even the same phytoconstituent could exhibit multiple mechanisms of action, as exemplified by curcumin and resveratrol [18].

The isolation and structural characterization of two new (**1**–**2**) and three known (**3**–**5**) labdanes, together with four flavonoids from *P. resinosa*, are reported. In more detail, a combined density functional theory (DFT)/NMR computational approach was applied to suggest the correct stereo assignment of these specialized metabolites, which represents a well-established method for supporting the structure elucidation of natural products [19,20].

The ATP-dependent molecular chaperone Hsp70 is over-expressed in cancer cells, where it is involved in the stabilization of onco-proteins, promoting cell proliferation and protecting cells from apoptosis and necrosis; for these reasons, Hsp70 is a promising target for cancer therapy [21,22,23]. Recently, the plant diterpenes oridonin and epoxysiderol were found to efficiently target Hsp70 1A in cancer cells; thus, the ability of the labdanes (**1**–**5**) to interact with the molecular chaperone Hsp70 was tested by a Surface Plasmon Resonance-screening [24].

Compound **3** was the most active. The potential interactions of the selected abietane with the Hsp70 ATP binding site were explored using molecular docking and molecular dynamic simulations.

## 2. Results

### 2.1. Phytochemical Investigation

**Figure 1:**
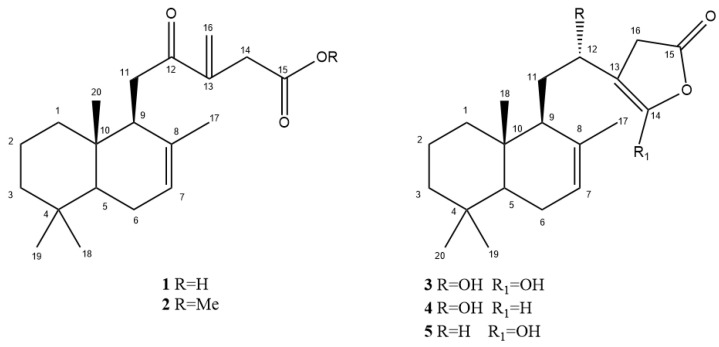
Structures of **1**–**5** isolated from *P. resinosa*.

The phytochemical investigation of the dichloromethane extract of the *P. resinosa* surface, after submission to silica gel column chromatography and RP-HPLC (obtaining seven fractions A–G, as reported in Section 4.3), afforded five diterpenes, two of which (**1**–**2**) were new natural compounds (premnaresone A and B) (Figure 1).

The structure of known compounds was characterized through NMR and mass spectrometry experiments and their comparison with literature data.

Known compounds were characterized as ent-12,16-dihydroxylabda-7,13-dien-15,16-olide (**3**) [25], ent-12-hydroxylabda-7,13-dien-15,16-olide (**4**) [25], ent-16-hydroxylabda-7,13-dien-15,16-olide (**5**) [26], myricetin 3,7,30-trimethyl ether [4], 3,7-dimethoxy quercetin [4], naringenin [27], and 5,4′-dihydroxy-6,7,8-trimethoxyflavone (xanthomicrol) [28].

**Figure 2:**
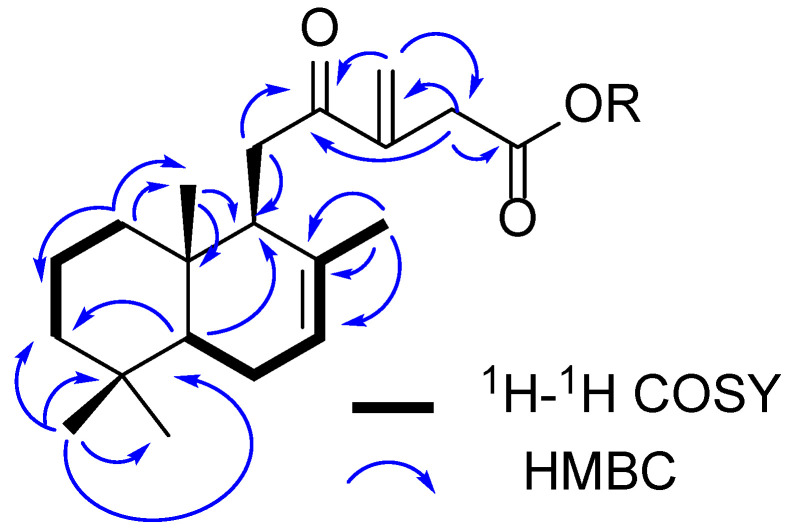
^1^H-^1^H COSY and HMBC correlation of **1** and **2**.

**1** was observed as a white, amorphous solid. The molecular formula of C_20_H_30_O_3_ was obtained for **1**, studying its HRESIMS data (*m*/*z* calculated [M+H]^+^ 319.2267; found 319.2262). The ^1^H NMR spectrum of **1** displayed signals for four methyl groups (δ_H_ 0.87 (s, Me-20), 0.93 (Me-18), 0.96 (s, Me-19), and 1.46 (s, Me-17)), one isolated methylene (δ_H_ 3.30 (d, *J* = 14.0, H_2_-14)), and three olefinic protons (δ_H_ 5.43, m, H-7; 5.94, s, 6.29, s, H_2_-16), which suggested that **1** is a labdane diterpenoid [29]. COSY correlations were monitored between the signals at δ_H_ 5.43 (H-7) and δ_H_ 2.05 and 1.96 (H_2_-6). The doublet multiplicity of the signal at δ_H_ 3.30 (H_2_-14) and the absence of COSY cross peaks showed that the corresponding protons were placed near non-protonated carbons. The other connectivity of the diterpenoid skeleton and the other functional groups was established from its ^1^H-^1^H COSY, HSQC, and HMBC spectra (Table 1 and Figure 2).

**Figure 3:**
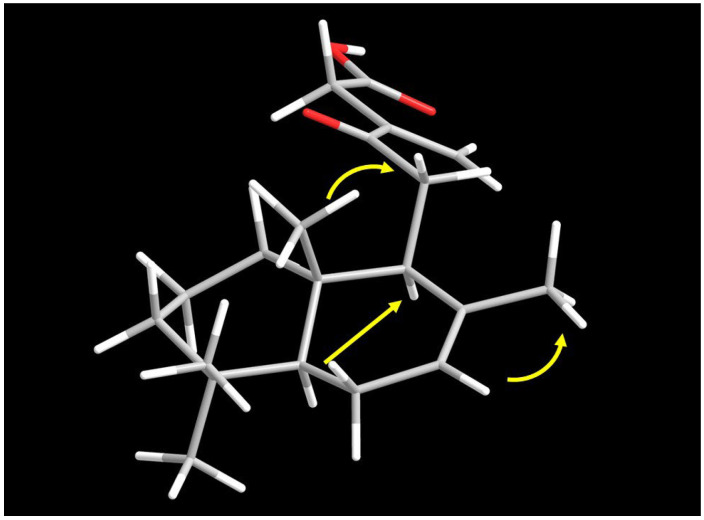
NOESY correlation of **1**.

The relative configuration of **1** was determined by studying ^1^H NMR coupling constants and NOESY correlations (Figure 3). The NOESY correlations between H-5 and H-9 and between Me-18 and Me-20 made it possible to hypothesize the relative stereochemistry of **1**. Thus, the structure of **1** could be proposed as 12-oxo-7,13(16)-labdandien-15-oic acid, and this compound has been accorded the trivial name, premnone D. 

The determination of the relative configuration of C-9 of **1** was performed through the approach based on the combination of QM/NMR [19,30,31]. Specifically, this methodology, developed and optimized by us [19,20], relies on the prediction of NMR parameters (e.g., ^13^C and ^1^H NMR chemical shift) through calculations based on the density functional theory (DFT). In more detail, the comparison of both predicted and experimental NMR parameters, through a statistical analysis, is the crucial point for suggesting the stereochemical assignment of organic and natural compounds [19,20,30]. The first step involved a wide-ranging conformational search of all the possible investigated stereoisomers of **1** by applying empirical methods, i.e., Monte Carlo Molecular Mechanics (MCMM), Low-Mode Conformational Sampling (LMCS), and Molecular Dynamics (MD) simulations (see computational details, Experimental Section). Specifically, the relative configuration of C-5 and C-10 has been assigned as S* and S*, respectively, since, in the literature, molecules with this core are characterized by *trans* arrangements considering the H-5 and Me-10. For this reason, two stereoisomers (**1a**–**1b**, Figure S14, Supporting Information) were considered for the prediction of chemical shifts. Afterwards, the MM conformer ensembles represented the input for the subsequent steps, involving quantomechanical geometry and energy optimization at the MPW1PW91/6-31g(d) level of theory. Eventually, the resulting optimized conformers were analyzed by visual inspection to eliminate further redundant conformers. Then, considering the Boltzmann distribution of the selected conformers for each investigated stereoisomer, ^13^C and ^1^H NMR chemical shifts were computed at the MPW1PW91/6-31g(d,p) level for **1a**–**b**. For all the DFT calculations, the simulation of the solvent methanol was performed using the integral equation formalism model (IEFPCM) [20,30,32].

Subsequently, to compare the calculated and experimental value, the statistical parameter mean absolute error (MAE) was used (see computational details, Experimental Section). Finally, **1b** featured the lowest ^13^C and ^1^H MAE values, i.e., 2.4 and 0.11 ppm, respectively (Tables S1 and S2), suggesting 5S*, 9S*, and 10S* as relative configurations for **1**. Moreover, to corroborate the obtained results, we also employed the DP4+ method [33], a robust tool for performing the correct stereochemical assignment of organic compounds. Accordingly, **1b** showed the highest DP4+ probabilities (100.0%), confirming the relative configuration 5S*, 9S*, and 10S*.

The HRESIMS spectrum of **2** showed the [M+Na]^+^ ion at *m*/*z* 355.2242, and ^13^C NMR showed 21 carbon signals. The multiplicities of carbons determined by ^13^C NMR data led to the attribution of four methyl groups, one methoxy group, seven methylenes, one of which sp^2^ hybridized, three methines, and two quaternary carbons, allowing for the proposal of the molecular formula C_21_H_32_O_3_ and a labdane diterpene. The NMR spectra (Table 1) of **2** were very similar to those of **1**, except for the presence of an ester functionality at C-17. Therefore, **2** was characterized as 12-oxo-7,13(16)-labdandien-15-oic acid methyl ester.

The relative configuration of **3** was 12*R*,16-dihydroxylabda-7,14-dien-15,16-olide, determined as reported in the literature [25,34,35,36].

Concerning **4**, four stereoisomers (**4a**–**4d**, Tables S3 and S4, Supporting Information) were considered for the prediction of chemical shifts. In this case, **4a** showed the lowest ^13^C and ^1^H MAE values (2.2 and 0.13 ppm, respectively). Accordingly, the DP4+ method was employed, highlighting 5S*, 9S*, 10S*, and 12R* as the relative configuration for **4** (DP4+ probabilities = 97.83%). It is worth noting that, through the application of the DFT/NMR approach for compounds **1** and **4**, only the relative configurations of the investigated compounds were suggested.

The relative configuration of **5** was 16-hydroxylabda-7,13-dien-15,16-olide, determined as reported in the literature [26,36].

### 2.2. Surface Plasmon Resonance (SPR)

**Figure 4:**
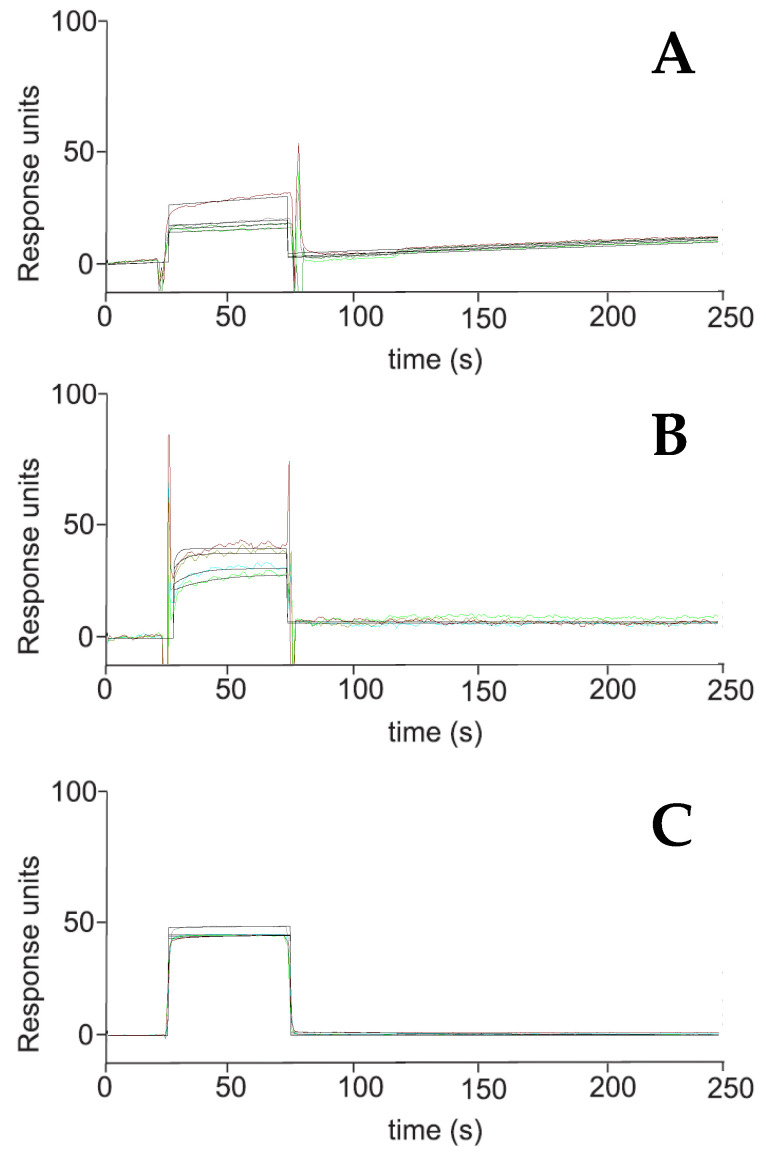
Surface plasmon resonance sensorgrams acquired for **3** (**A**) interacting with Hsp70 and for the positive control oridonin (**B**) and **5**, a non-binding compound (**C**). Each compound was injected onto an Hsp70 modified sensor chip at six (n = three) different concentrations in the range 0.025–4.000 µM.

Our previous results suggest that diterpenes could modulate the Hsp70 activity. **1**–**5** were studied by Surface Plasmon Resonance (SPR) experiments to investigate their interaction with this chaperone; oridonin, a well-known Hsp70 inhibitor, was used as a positive control. The interaction between each of the labdanes (**1**–**5**) and Hsp70 was investigated in this study by a surface plasmon resonance (SPR)-based binding assay [24,37,38,39]. Only **3** interacted efficiently with the immobilized protein. As a result of fitting the relative sensorgrams to a single-site bimolecular interaction model, the thermodynamic parameters for the resulting complex formation were determined. This approach allowed for the measurement of 90.8 ± 9.5 nM K_D_ for the Hsp70/**3** complex. Interestingly, **3** showed a similar affinity towards the chaperone compared with that determined for oridonin (Table 2, Figure 4). Instead, **4** showed a lower affinity than **3** (in order of micromolar), with a value of 1000 ± 25 nM.

Then, we evaluated the potential antiproliferative or cytotoxic activity of **1**–**5** on human HeLa (epithelial carcinoma) and Jurkat (human T-cell lymphoma) cell lines. The cells were incubated for 48 h with increasing concentrations of labdane (5–50 µM), and cell viability was determined by an MTT proliferation assay [40,41]. Among those tested, **5** was inactive, while **3**–**4** showed low activity on Jurkat cells and were not effective towards HeLa cells. **1** showed moderate activities on both cell lines. **3** demonstrated an interesting antiproliferative activity, showing an IC_50_ value of 15.2 ± 1.0 µM in the Jurkat cell line (Table 3).

### 2.3. Computational Analyses

Molecular docking calculation and molecular dynamics were applied to investigate the binding of Hsp70 and **3**. The Protein Data Bank [42] entry 5AQX was used to represent the structure of the enzyme and to explore the binding of possible inhibitors at the ATP binding site of the protein [43]. 

Glide [44] was used for docking simulation, focusing on the ATP binding site of the protein; the analysis of binding poses reported a hydrogen bond network between Hsp70 and **3**. Specifically, the ligand was bound with Thr13, Thr14, and Gly339 amino acid residues in the binding cavity (Figure S15, Supporting Information). The selected ligand reported a predicted binding affinity value of −6.258 (kcal/mol; Glide XP), with Hsp70 [45]. Then, 100 ns of the molecular dynamic simulation were carried out to evaluate the stability of the complex during the whole simulation time, and the results can be visualized in the protein–ligand RMSD plot (Figure S16A, Supporting Information). The in silico investigation highlighted the stability of the binding during the whole simulation. The “Lig fit Prot” plot shows that the RMSD of the ligand is lower than the RMSD of the protein, underlining that the ligand is stable in its initial binding site. The protein–ligand contacts over time provided interesting information about the binding behavior of the complex. Interactions of **3** with the key residues Thr13 (H-bonds and water bridges) and Ser240, Gly202, and Lys271 were detected. Gly202 has been reported to interact with the α-helix at the back of the ATP binding site [43]. Thr13 is close to Thr15, a critical residue known to stabilize the protein’s close conformation [24,43]. The residues around Arg275 have been indicated as selective determinants in the initial ATP-binding. **3** showed several hydrogen-bonded protein–ligand interactions mediated by a water molecule, including Arg72, Glu175, Gly202, and Gly339 (Figure S16B, Supporting Information).

## 3. Discussion

The literature data reported an increasing number of Hsp70 inhibitors, acting with different mechanisms analyzed using chemical and biological approaches (for example, surface plasmon resonance, chemical proteomics, computational details, interaction on HSP70 cochaperones that modulate the chaperone activity of the protein through their binding to functional domains of Hsp70, Hsp70 ATPase activity test, regulation of Hsp70 client proteins, citrate synthase aggregation assay, co-immunoprecipitation experiments) [26,46,47,48,49,50,51,52,53,54]. The investigation of the protein–ligand complex structure and behavior is fundamental in the drug discovery process. In a previous study, the treatment of leukemia-derived Jurkat cells with oridonin inhibited Hsp70 [55]. **3** stably interacted with Hsp70 efficiently compared with oridonin, as confirmed by the SPR and molecular modeling studies. The SPR-based binding assay results in this study showed the efficient interaction of **3** with the Hsp70 protein at 91 nM K_D_, which was similar to the chaperone compared with that determined for oridonin (positive control). This result was supported by an in silico investigation, which showed that **3** was accurately placed into the Hsp70 ATP binding site. In addition, 100 ns of molecular dynamic simulation reported several crucial interactions involved in the stabilization of the studied complex. Most recently, the kaurane diterpene ent-7*β*-acetoxy-18-hydroxy-15*α*,16*α*-epossikaurane (epoxysiderol) has been reported as a modulator of Hsp70 1A activity [24]. Particularly, **3** reported a similar hydrogen bonds pattern of epoxysiderol, involving different key residues for developing Hsp70 inhibitors. Moreover, epoxysiderol reported a similar binding affinity value of **3**, interacting in the same ATP binding site [24]. All these findings suggest the molecular explanation of the experimentally detected binding affinity, suggesting **3** as a new possible Hsp70 inhibitor.

Molecular docking simulations were also performed for **1**, **2**, **4**, and **5**, which cannot bind the active site of Hsp70, in accordance with the biophysical data for SPR experiments. Further studies will be performed to identify natural compounds targeting Hsp70 1A in cancer cells.

Plants represent a remarkable reservoir of biomolecules exhibiting different biological activities. However, one major challenge in phytochemical studies lies in the intricate process of isolating and characterizing the active constituents. Often, these phytoconstituents are obtained in minute quantities, which hinders further investigations. Nonetheless, these limitations can be overcome through interdisciplinary collaborations that focus on producing active constituents via plant tissue and cell culture techniques [56]. Alternatively, chemical synthesis offers a viable and accessible approach for synthesizing biomolecules with exceptional bioactivity [57]. Moreover, the utilization of sophisticated nano-formulations holds promise in achieving the enhanced targeting of tumoral masses [58,59].

## 4. Materials and Methods

### 4.1. General

An Atago AP-300 digital polarimeter with a 1 dm microcell and a sodium lamp (589 nm) was used to obtain optical rotations. A Bruker DRX-600 NMR spectrometer (Bruker BioSpin GmBH, Rheinstetten, Germany) equipped with a Bruker 5 mm TCI at 300 K was employed to run NMR data. Data processing was carried out with Topspin 3.2 software. All 2D NMR spectra were acquired in methanol-d_4_ (99.95%, Sigma-Aldrich, Milano, Italy), and standard pulse sequences and phase cycling were used for COSY, HSQC, HMBC, 1D-TOCSY, and ROESY spectra. HRESIMS data were obtained in the positive ion mode on a Q Exactive Plus mass spectrometer, an Orbitrap-based FT-MS system, equipped with an ESI source (Thermo Fischer Scientific Inc., Bremen, Germany). Column chromatography was performed over silica gel (70–220 mesh, Merck). A Shimadzu LC-8A series pumping system equipped with a Shimadzu RID-10A refractive index detector and Shimadzu injector (Shimadzu Corporation, Kyoto, Japan) on a Waters XTerra Semiprep MS C_18_ column (300 mm × 7.8 mm i.d.) and a mobile phase consisting of a MeOH-H_2_O mixture at a flow rate of 2.0 mL/min were employed to purify the molecules. TLC was performed on silica gel 60 F_254_ (0.20 mm thickness) plates (Merck, Darmstadt, Germany) as a spray reagent, and Ce(SO_4_)_2_/H_2_SO_4_ (Sigma-Aldrich, Milano, Italy) was used [60].

### 4.2. Plant Material

*Premna resinosa* (Hochst.) Schauer (Lamiaceae) leaves were collected at Al-Kurr, Makkah Province, Saudi Arabia, in June 2018 and identified by one of the authors, Prof. A. Bader. A voucher specimen was deposited at the Herbarium of Pharmacognosy Lab at the Faculty of Pharmacy, Umm Al-Qura University (n. UQU-SA-109).

### 4.3. Extraction and Isolation

The exudate of *P. resinosa* (2.1 g) was obtained by dipping 200 g of fresh leaves into 2.1 L of dichloromethane for less than 30 s and was dried at 40 °C. The dichloromethane extract was dissolved in CHCl_3_ and subjected to silica gel CC eluting with CHCl_3_, followed by increasing concentrations of CH_3_OH in CHCl_3_ (between 1% and 100%) and collecting seven major fractions (A–G). 

Fraction B (128.4 mg) was subjected to RP-HPLC on a C18 µ-Bondapak column with MeOH-H_2_O (7:3) as the eluent to give 3,7-di methoxy quercetin (2.2 mg, t_R_ 10 min) and myricetin 3,7,3′-trimethyl ether (2.0 mg, t_R_ 15 min). 

Fraction C (80.0 mg) was purified by RP-HPLC on a C18 µ-Bondapak column with MeOH-H_2_O (7:3) as the eluent to give **3** (0.5 mg, t_R_ 20 min), **4** (1.0 mg, t_R_ 28 min), and **5** (9.3 mg, t_R_ 36 min).

Fraction D (321.0 mg) was subjected to RP-HPLC on a C18 µ-Bondapak column with MeOH-H_2_O (4:1) as the eluent to give naringenin (2.2 mg, t_R_ 6 min) and xanthomicrol (1.7 mg, t_R_ 16 min).

Fractions E (15.3 mg) and F (46.2 mg) were purified by RP-HPLC on a C18 µ-Bondapak column with MeOH-H_2_O (4:1) as the eluent to give **3** (1.1 mg, t_R_ 15 min) from E and **1** (1.5 mg, t_R_ 25 min) and **2** (1.8 mg, t_R_ 33min) from F.

12-oxo-7,13(16)-labdandien-15-oic acid (premnaresone A) (**1**). Amorphous white powder; m.p. 94–97 °C; ${\left[\alpha \right]}_{D}^{25}$: +22.3 (c 0.06, CH_3_OH); ^1^H NMR (CD_3_OD, 600 MHz) and ^13^C NMR (CD_3_OD, 150 MHz); see Table 1; HRESIMS *m*/*z* 319.2262 [M+H]^+^, calcd. for C_20_H_31_O_3_, 319.2267.

12-oxo-7,13(16)-labdandien-15-oic acid methyl ester (premnaresone B) (**2**). Amorphous white powder; m.p. 95–98 °C; ${\left[\alpha \right]}_{D}^{25}$: +22. 8 (c 0.06, CH_3_OH); ^1^H NMR (CD_3_OD, 600 MHz) and ^13^C NMR (CD_3_OD, 150 MHz); see Table 1; HRESIMS *m*/*z* 355.2242 [M+Na]^+^, calcd. for C_21_H_32_O_3_Na, 355.2243.

### 4.4. Surface Plasmon Resonance Analyses (SPR)

To investigate the interaction between **3** and Hsp70, the surface plasmon resonance (SPR) analyses were performed using a Biacore 3000 optical biosensor equipped with research-grade CM5 sensor chips (GE Healthcare, Chicago, IL, USA), according to a previously detailed method [38]. Recombinant Hsp70 surfaces, a BSA surface, and an unmodified reference surface were prepared. Proteins (100 μg/mL in 10 mM sodium acetate, pH 5.0) were immobilized on individual sensor chip surfaces at a flow rate of 5 μL/min to produce densities of 8−12 kRU. **3**, **4**, and oridonin (positive control) were dissolved in DMSO. The six-point concentration series (0.025−4.000 μM) was prepared. Bioevaluation software (GE Healthcare) was used to elaborate simple interactions, which were adequately fit to a single-site bimolecular interaction model (A + B = AB), yielding a single K_D_ sensorgram [37,61].

### 4.5. Cell Culture and Treatment

HeLa (cervical carcinoma) and Jurkat (T-cell lymphoma) cell lines were purchased from the American Type Cell Culture (ATCC) (Rockville, MD, USA). The cells were cultured in DMEM (HeLa) or RPMI 1640 (Jurkat), supplemented with 10% FBS, 100 mg/L streptomycin, and 100 IU/mL penicillin, at 37 °C in a humidified atmosphere of 5% CO_2_. Stock solutions of compounds were prepared in DMSO (50 mM) and stored in the dark at 4 °C. In all experiments, the final concentration of DMSO did not exceed 0.15% (*v*/*v*) [62].

### 4.6. Cell Viability

Cell viability was evaluated by an MTT ([3-(4,5-dimethylthiazol-2-yl)-2,5-diphenyltetrazolium bromide]) assay. Briefly, the cells were plated in 96-well tissue culture plates (7.5 × 10^3^ cells/well for Hela and 4 × 10^5^ for Jurkat). After 24 h, serial dilutions of **1**–**5** (10–100 µM) were incubated for 48 h. Then, MTT was incubated at the final concentration of 1 mg/mL and incubated for an additional 3 h to allow for the formation of purple formazan precipitate; then, 100 μL of lysis buffer solution (50% *v*/*v*) *N*,*N*-dimethylformamide and 20% (*w*/*v*) SDS with an adjusted pH of 4.5 were added. The optical density (OD) of each well was measured with a microplate spectrophotometer (Multiskan Spectrum).

### 4.7. Computational Details

All the steps described in this paragraph were accomplished for all the stereoisomers investigated. For the generation of the starting 3D structure of **1** and **4**, Maestro 12.7 was applied, and then, MacroModel 13.1 was employed for the optimization of the 3D structures, using the OPLS force field and the Polak-Ribier conjugate gradient algorithm (PRCG, maximum derivative less than 0.001 kcal/mol).

In more detail, for **1**, featuring three stereogenic centers, two of which were assigned as S* (for C-5) and S* (for C-10), two possible isomers were considered: 

**1a** (5S*, 9R*, 10S*), **1b** (5S*, 9S*, 10S*) (Figure S14, Supporting Information).

For **4**, showing four stereogenic centers, two of which were assigned as S* and S* (at C-5 and C-10, respectively), four isomers were considered:

**4a** (5S*, 9S*, 10S*, 12R*), **4b** (5S*, 9S*, 10S*, 12S*), **4c** (5S*, 9R*, 10S*, 12S*), **4d** (5S*, 9R*, 10S*, 12R*) (Figure S14, Supporting Information).

Subsequently, wide-ranging conformational search cycles at the empirical molecular mechanics (MM) level were carried out, performing Monte Carlo Multiple Minimum (MCMM) (50,000 steps) and Low Mode Conformational Search (LMCS) rounds (50,000 steps). 

In addition, MD simulations of 10ns were performed, setting different temperatures (i.e., 450, 600, 700, and 750 K), a time step of 2 fs, an equilibration time of 0.1 ns, and a constant dielectric term of methanol to consider the presence of the solvent.

The minimization step (PRCG, maximum derivative less than 0.001 kcal/mol) of the sampled conformers was performed, and, using the “Redundant Conformer Elimination” MacroModel 13.1+, all the conformers differing by more than 21.0 kJ/mol (5.02 kcal/mol) from the most energetically favored and a 0.5 Å RMSD (root-mean-square deviation) were saved for the next steps. Indeed, the non-redundant conformers represented the input for QM calculations with Gaussian 09 software [32]. The MPW1PW91 functional and the 6–31G (d) basis set were employed for the conformer’s geometry optimization step at the DFT level [63]. The integral equation formalism version of the polarizable continuum model (IEFPCM) related to MeOH was used for the solvent simulation [64]. To ensure the absence of further possible redundant conformers, the optimized geometries were also analyzed by visual inspection. The MPW1PW91 functional, the 6–31G (d,p) basis set, and methanol IEFPCM were used to compute the ^13^C and ^1^H NMR chemical shifts of each conformer of each of the accounted isomers of **1** and **4**. Eventually, final ^13^C and ^1^H NMR datasets were obtained, considering the energy-based weight of each conformer on the total Boltzmann distribution.

The calibration of calculated chemical shifts was carried out, accounting for a multi-standard approach (MSTD) [65] and according to this procedure: sp^2 13^C and ^1^H NMR chemical shifts were computed, taking benzene as reference [65], while sp^3 13^C and ^1^H chemical shifts were calibrated considering TMS (Tables S1–S4, Supporting Information).

In parallel, for the computation of the DP4+ probabilities, a further dataset was obtained, only considering TMS as reference (Tables S1, S2, S5 and S6, Supporting Information).

The final comparison of the experimental and predicted NMR data was accomplished through the Δδ parameter (Tables S1–S4, Supporting Information):

Δδ = |δ_exp_ − δ_calc_|

where δ_exp_ (ppm) refers to ^13^C/^1^H experimental chemical shifts and δ_calc_ (ppm) refers to calculated shifts.

Subsequently, the mean absolute error (MAE) values for the final statistical analysis were computed as follows:

$$MAE=\frac{\sum \left(\Delta \delta \right)}{n}$$

i.e., the summation (Σ) of all calculated absolute error values (Δδ), normalized considering the number of chemical shifts (n) (Tables S1–S4, Supporting Information).

The DP4+ probabilities for each investigated stereoisomer of **1** and **4** were obtained, taking into account the chemical shift data obtained by using only TMS as the reference compound (Tables S1–S4, Supporting Information) and then manually selecting the sp^2^ atoms in the available DP4+ Toolbox (Excel file).

### 4.8. Molecular Docking

The 3D chemical structures of **1**–**5** were built using Maestro v. 11.1 (Schrödinger Suite 2020-4) [44]. The generated structures were prepared using LigPrep software (Schrodinger Suite) [44] and were then minimized using an OPLS3e force field. For each ligand, all possible tautomers and the protonation state at a pH of 7.0 ± 2.0 were enumerated. 

The protein 3D structure was prepared using the Schrödinger Protein Preparation Wizard [44], starting from the Hsp70 X-ray structure co-complexed with the inhibitor KC7 (PDB code: 5AQX). Water molecules were removed, all hydrogen atoms were added, and bond orders were assigned. The grid box was placed on the co-complexed ligand using the Receptor Grid Generation tool [44]. Molecular docking experiments were performed using Glide software (also part of the Schrödinger Suite) [44] and the Standard Precision (SP) and Extra Precision (XP) scoring/sampling mode.

### 4.9. Molecular Dynamics

The XP docked pose of **3** bound to Hsp70 (PDB code: 5AQX) was submitted to molecular dynamics simulations using the Desmond Molecular Dynamic [44]. The starting complex was prepared by the System Builder in Desmond. A cubic box with a 10 Å buffer distance was set; the TIP3P water model for solvation and the OPLS3e force field were employed, and 16 Na^+^ ions were added for obtaining the electroneutrality. The built systems were then minimized by the Minimization tool in Desmond. MD simulations of 100 ns at 310 K, using a recording interval of 100 ps and an ensemble class NPT (1.01 bar), were then performed.

## 5. Conclusions

Our study has provided valuable insights into the phytochemical constituents of *P. resinosa*—specifically, its exudated metabolites. These compounds hold great potential for further exploration, not only within the same genus but also in other plants that contain similar classes of compounds. Utilizing cheminformatics, we successfully identified the active pharmacophore and elucidated the mechanism of action of the isolated labdane diterpenes. Future studies should focus on developing sophisticated micro- and nanoformulations to optimize therapeutic dosing and enable targeted delivery to diseased organs. Additionally, plant tissue and cell culture approaches and chemical synthesis may overcome the limitations associated with the isolation and characterization of bioactive constituents present in trace amounts. By collaborating across different disciplines, we can harness the diverse biological activities of plant-derived biomolecules and accelerate the development of effective anticancer therapies.

## Figures and Tables

**Table 1:** ^1^H and ^13^C NMR Spectroscopic data for **1** and **2** (CD_3_OD; 600.23 MHz for ^1^H and 150.93 MHz for ^13^C NMR).

	1			2		
Position	*δ*_C_, Type	*δ*_H_	HMBC	*δ*_C_, Type	*δ*_H_	HMBC
1	38.7, CH_2_	1.74, brd (14.0) 1.01, ddd (16.0, 13.0, 3.0)	2, 5, 10, 20	38.7, CH_2_	1.74, brd (14.0) 1.01, ddd (16.0, 13.0, 3.0)	2, 5, 10, 20
2	18.0, CH_2_	1.50, m 1.46 ^a^	1, 10	18.0, CH_2_	1.50, m ^LAKI^ 1.46 ^a^	1, 10
3	41.7, CH_2_	1.46 ^a^ 1.24, ddd (17.3, 14.0, 4.0)	4, 5, 19	41.7, CH_2_	1.46 ^a^ 1.24, ddd (17.3, 14.0, 4.0)	4, 5, 19
4	33.0, C	-	-	33.0, C	-	-
5	50.1, CH	1.33, dd (12.0, 5.0)	1, 4, 6, 9, 18, 19, 20	50.1, CH	1.33, dd (12.0, 5.0)	1, 4, 6, 9, 18, 19, 20
6	23.5, CH_2_	2.05, brd (16.0) 1.96, brt (16.0)	8	23.5, CH_2_	2.05, brd (16.0) 1.96, brt (16.0)	8
7	122.0, CH	5.43, m	5	122.0, CH	5.43, m	5
8	134.0, C	-	-	134.0, C	-	-
9	48.6, CH	2.71, brd (8.5)	8	48.6, CH	2.71, brd (8.5)	8
10	36.0, C	-	-	36.0, C	-	-
11	35.0, CH_2_	2.94, dd (18.3, 8.7) 2.63, brd (18.3)	8, 9, 12	35.0, CH_2_	2.94, dd (18.3, 8.7) 2.63, brd (18.3)	8, 9, 12
12	203.0, C	-	-	203.0, C	-	-
13	144.0, C	-	-	144.0, C	-	-
14	37.2, CH_2_	3.30, d. (14.0) 3.30, d. (14.0)	12, 13, 15, 16	37.2, CH_2_	3.30, d. (17.5) 3.30, d. (17.5)	12, 13, 15, 16
15	174.5, C	-	-	173.4, C	-	-
16	125.5, CH_2_	6.30, brs 5.94, brs	12, 13, 14	125.5, CH_2_	6.30, brs 5.94, brs	12, 13, 14
17	21.1, CH_3_	1.46, s	7, 8, 9	21.1, CH_3_	1.46, s	7, 8, 9
18	32.0, CH_3_	0.93, s	3, 4, 5, 18	32.0, CH_3_	0.93, s	3, 4, 5, 18
19	21.0, CH_3_	0.96, s	3, 4, 5, 19	21.0, CH_3_	0.96, s	3, 4, 5, 19
20	13.0, CH_3_	0.87, s	1, 9, 10	13.0, CH_3_	0.87, s	1, 9, 10
MeO	-	-	-	51.0, CH_3_	3.68, s	

^a^ Overlapped signal.

**Table 2:** Thermodynamic constants (mean ± sd) measured by SPR for the interaction between the tested compounds and immobilized Hsp70.

Compound	K_D_ (nM) ^a^
**1**	No binding
**2**	No binding
**3**	90.8 ± 3.5
**4**	1000 ± 25
**5**	No binding
Oridonin	81.4 ± 12.4

^a^ Results were given as the mean ± standard deviation.

**Table 3:** IC_50_ (μM) values of tested compounds against two cancer cell lines ^a^.

Compound	Jurkat ^b^	HeLa ^c^
**1**	25.1 ± 1.0	24.5 ± 1.7
**2**	20.8 ± 1.2	>50
**3**	15.2 ± 1.0	>50
**4**	42.5 ± 1.4	>50
**5**	>50	>50
**Etoposide**	2.5 ± 0.4	4.0 ± 0.8

^a^ Mean values ± SD from three experiments conducted in triplicate; ^b^ T-cell lymphoma; ^c^ Cervical carcinoma.

## Data Availability

The data presented in this study are available within this article.

## Supplementary Materials

The following supporting information can be downloaded at: https://www.mdpi.com/article/10.3390/plants12132421/s1, Figure S1: 1H NMR spectrum of **1** (CD_3_OD, 600 MHz); Figure S2: 13C NMR spectrum of **1** (CD_3_OD, 600 MHz); Figure S3: COSY spectrum of **1** (CD_3_OD, 600 MHz); Figure S4: HSQC spectrum of **1** (CD_3_OD, 600 MHz); Figure S5: HMBC spectrum of **1** (CD_3_OD, 600 MHz); Figure S6: NOESY spectrum of compound **1** (CD_3_OD, 600 MHz); Figure S7: HRESIMS of **1**; Figure S8: 1H NMR spectrum of **2** (CD_3_OD, 600 MHz); Figure S9: 13C NMR spectrum of **2** (CD_3_OD, 600 MHz); Figure S10: COSY spectrum of **2** (CD_3_OD, 600 MHz); Figure S11: HSQC spectrum of **2** (CD_3_OD, 600 MHz); Figure S12: HMBC spectrum of **2** (CD_3_OD, 600 MHz); Figure S13: HRESIMS of **2**; Figure S14: 2D structures of investigated stereoisomers of **1** and **4**; Figure S15: Binding pose and interaction of **3** docked to the Hsp70 ATP binding site; Figure S16: Molecular dynamic simulation results; Table S1: 1H experimental and calculated NMR chemical shifts for **1a**–**b**, with ^a^|Δδ|(^1^H) and ^b^MAE values. The chemical shift data reported here were produced using benzene as the reference compound for sp^2^ hydrogens and tetramethylsilane (TMS) for sp^3^ hydrogens; Table S2: 13C experimental and calculated NMR chemical shifts for **1a**–**b**, with ^a^|Δδ|(^13^C) and ^b^MAE values. The chemical shift data reported here were produced using benzene as a reference compound for sp^2^ carbons and tetramethylsilane (TMS) for sp^3^ carbons; Table S3: 1H experimental and calculated NMR chemical shifts for **4a**–**b**, with ^a^|Δδ|(^1^H) and ^c^MAE values. The chemical shift data reported here were produced using benzene as the reference compound for sp2 hydrogens and tetramethylsilane (TMS) for sp3 hydrogens; Table S4: 13C experimental and calculated NMR chemical shifts for **4a**–**b**, with ^a^|Δδ|(^13^C) and ^b^MAE values. The chemical shift data reported here were produced using benzene as the reference compound for sp^2^ carbons and tetramethylsilane (TMS) for sp^3^ carbons.
