# Supplementary material for: Exploring the Anticancer Potential of Premna resinosa (Hochst.) Leaf Surface Extract: Discovering New Diterpenes as Heat Shock Protein 70 (Hsp70) Binding Agents
Source: Plants (Basel). 2023 Jun 22;12(13):2421. doi: 10.3390/plants12132421 (PMC10347124; doi:10.3390/plants12132421)
Supplement: Supplementary file 1 [file plants-12-02421-s001.zip › plants-2428125-supplementary.pdf]

plants

*Supplementary Materials*

# Exploring the Anticancer Potential of *Premna resinosa* (Hochst.) Leaf Surface Extract: Discovering New Diterpenes as Heat Shock Protein 70 (Hsp70) Binding Agents

**Valentina Parisi [1], Giuliana Donadio [1], Maria Laura Bellone [1], Soumia Belaabed [2], Ammar Bader [3], Angela Bisio [4], Valeria Iobbi [4], Erica Gazzillo [1], Maria Giovanna Chini [5], Giuseppe Bifulco [1], Immacolata Faraone [6,7] and Antonio Vassallo [6,8,*]**

[1] Department of Pharmacy, University of Salerno, Via Giovanni Paolo II 132, 84084 Fisciano, Italy; vparisi@unisa.it (V.P.); gdonadio@unisa.it (G.D.); mbellone@unisa.it (M.L.B.); egazzillo@unisa.it (E.G.); bifulco@unisa.it (G.B.)
[2] Department of Chemistry, Research Unit, Development of Natural Resources, Bioactive Molecules Physicochemical and Biological Analysis, University Brothers Mentouri, Route Ain ElBey, Constantine 25000, Algeria; soumia.belaabed@umc.edu.dz
[3] Department of Pharmacognosy, Faculty of Pharmacy, Umm Al-Qura University, Makkah 21955, Saudi Arabia; ambader@uqu.edu.sa
[4] Department of Pharmacy, University of Genova, Viale Cembrano 4, 16148 Genova, Italy; bisio@difar.unige.it (A.B.); valeria.iobbi@edu.unige.it (V.I.)
[5] Department of Biosciences and Territory, University of Molise, C.da Fonte Lappone, 86090 Pesche, Italy; mariagiovanna.chini@unimol.it
[6] Department of Science, University of Basilicata, Viale dell'Ateneo Lucano 10, 85100 Potenza, Italy; immacolata.faraone@unibas.it
[7] Innovative Startup Farmis s.r.l., Via Nicola Vaccaro 40, 85100 Potenza, Italy
[8] Spinoff TNcKILLERS s.r.l., Viale dell'Ateneo Lucano 10, 85100 Potenza, Italy
* Correspondence: antonio.vassallo@unibas.it; Tel.: +39-0971205624

**Citation:** Parisi, V.; Donadio, G.; Bellone, M.L.; Belaabed, S.; Bader, A.; Bisio, A.; Iobbi, V.; Gazzillo, E.; Chini, M.G.; Bifulco, G.; et al. Exploring the Anticancer Potential of *Premna resinosa* (Hochst.) Leaf Surface Extract: Discovering New Diterpenes as Heat Shock Protein 70 (Hsp70) Binding Agents. *Plants* **2023**, *12*, 2421. https://doi.org/10.3390/10.3390/plants12132421

Academic Editor: Maria José U. Ferreira

Received: 16 May 2023
Revised: 12 June 2023
Accepted: 20 June 2023
Published: 22 June 2023

**Copyright:** © 2023 by the authors. Submitted for possible open access publication under the terms and conditions of the Creative Commons Attribution (CC BY) license (https://creativecommons.org/licenses/by/4.0/).

**Figure S1.** $^{1}H$ NMR spectrum of compound **1** ($CD_3OD$, 600 MHz).
**Figure S2.** $^{13}C$ NMR spectrum of compound **1** ($CD_3OD$, 600 MHz).
**Figure S3.** COSY spectrum of compound **1** ($CD_3OD$, 600 MHz).
**Figure S4.** HSQC spectrum of compound **1** ($CD_3OD$, 600 MHz).
**Figure S5.** HMBC spectrum of compound **1** ($CD_3OD$, 600 MHz).
**Figure S6.** NOESY spectrum of compound **1** ($CD_3OD$, 600 MHz)**Figure S7.** HRESIMS of compound **1**.
**Figure S8.** $^{1}H$ NMR spectrum of compound **2** ($CD_3OD$, 600 MHz).
**Figure S9.** $^{13}C$ NMR spectrum of compound **2** ($CD_3OD$, 600 MHz).
**Figure S10.** COSY spectrum of compound **2** ($CD_3OD$, 600 MHz).
**Figure S11.** HSQC spectrum of compound **2** ($CD_3OD$, 600 MHz).
**Figure S12.** HMBC spectrum of compound **2** ($CD_3OD$, 600 MHz).
**Figure S13.** HRESIMS of compound **2**.
**Figure S14.** 2D structures of investigated stereoisomers of **1** and **4**.
**Figure S15.** Binding pose and interaction of **3** docked to Hsp70 ATP binding site.
**Figure S16.** Molecular dynamic simulation results.

**Table S1.** $^{1}H$ experimental and calculated NMR chemical shifts for **1a**-**b**, with $^{a}|\Delta\delta|(^{1}H)$ and $^{c}$MAE values. Chemical shift data here reported were produced using benzene as reference compound for $sp^2$ hydrogens, and tetramethylsilane (TMS) for $sp^3$ hydrogens.

**Table S2.** $^{13}C$ experimental and calculated NMR chemical shifts for **1a**-**b**, with $^{a}|\Delta\delta|(^{13}C)$ and $^{b}$MAE values. Chemical shift data here reported were produced using benzene as reference compound for $sp^2$ carbons, and tetramethylsilane (TMS) for $sp^3$ carbons.

**Table S3.** $^{1}H$ experimental and calculated NMR chemical shifts for **4a**-**d**, with $^{a}|\Delta\delta|(^{1}H)$ and $^{c}$MAE values. Chemical shift data here reported were produced using benzene as reference compound for $sp^2$ hydrogens, and tetramethylsilane (TMS) for $sp^3$ hydrogens.

**Table S4.** $^{13}C$ experimental and calculated NMR chemical shifts for **4a**-**d**, with $^{a}|\Delta\delta|(^{13}C)$ and $^{b}$MAE values. Chemical shift data here reported were produced using benzene as reference compound for $sp^2$ carbons, and tetramethylsilane (TMS) for $sp^3$ carbons.

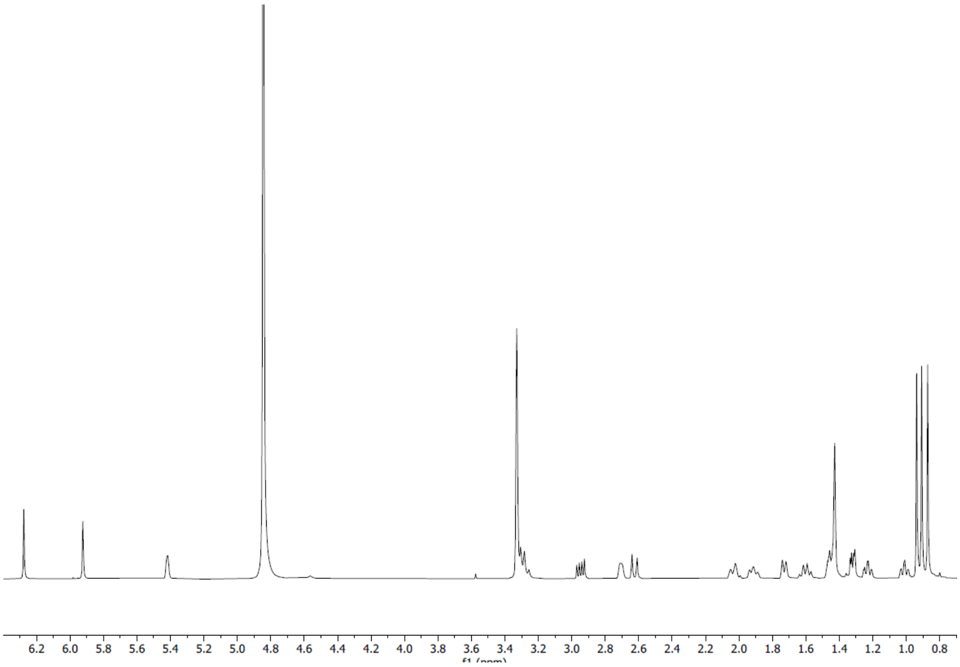


**Figure S1.** $^{1}$H NMR spectrum of compound **1** (CD3OD, 600 MHz).

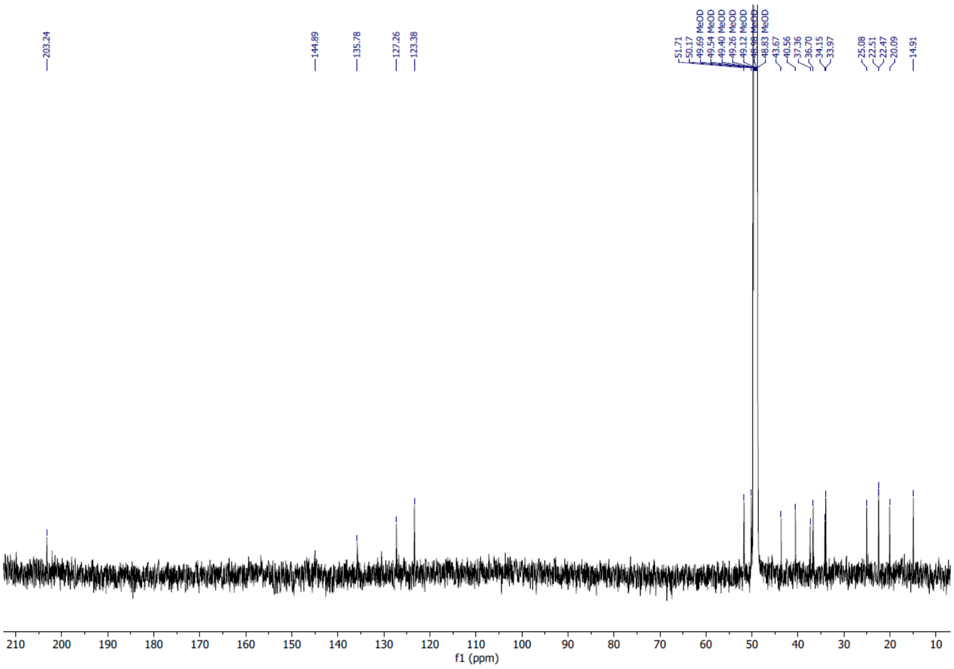


**Figure S2.** $^{13}$C NMR spectrum of compound **1** (CD3OD, 600 MHz).

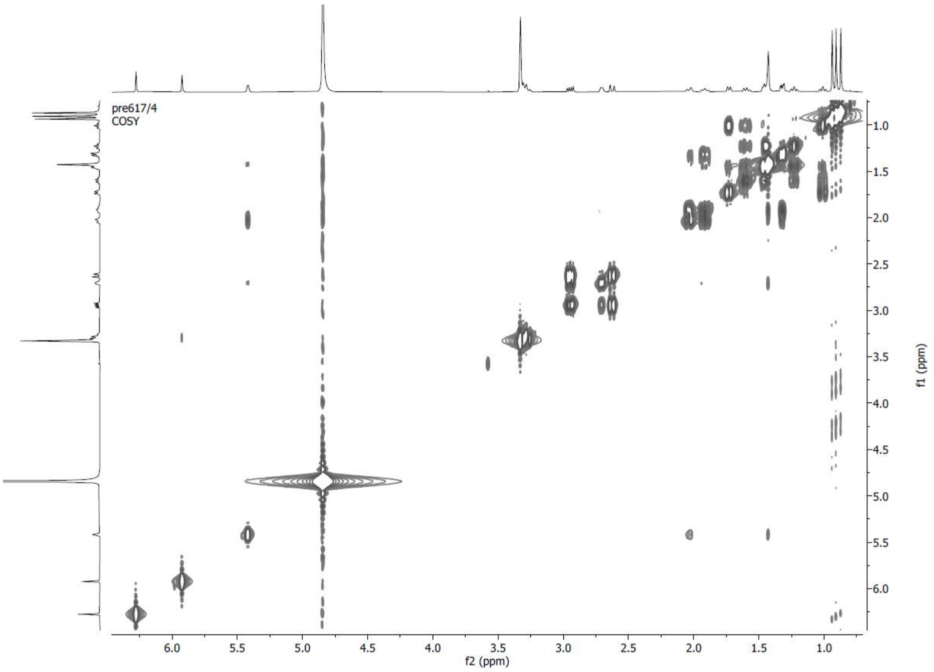


**Figure S3.** COSY spectrum of compound **1** ($CD_3OD$, 600 MHz).

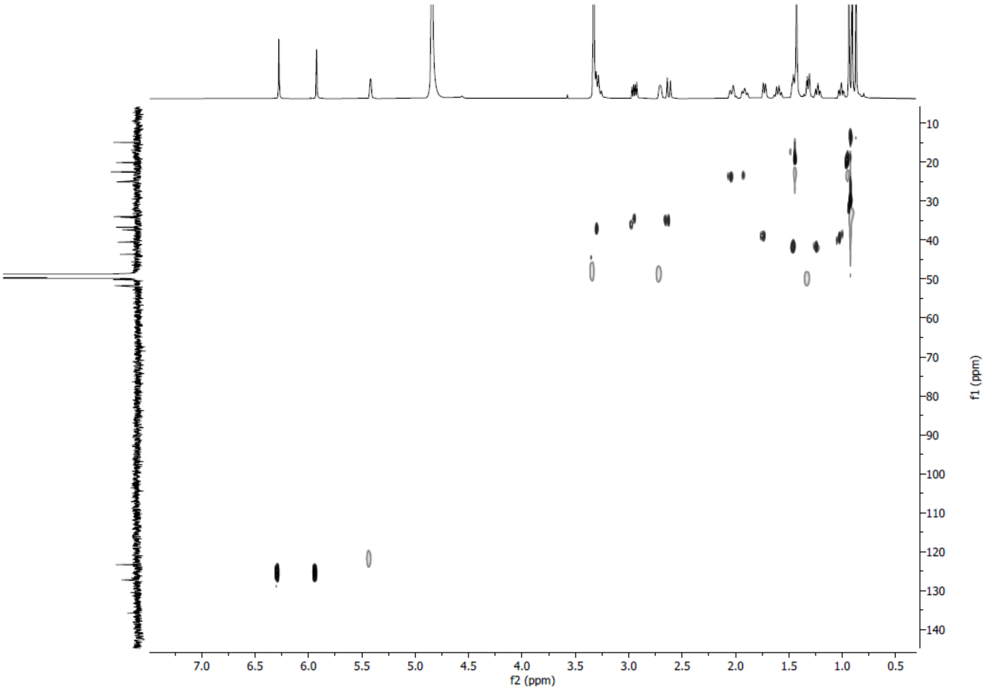


**Figure S4.** HSQC spectrum of compound **1** ($CD_3OD$, 600 MHz).

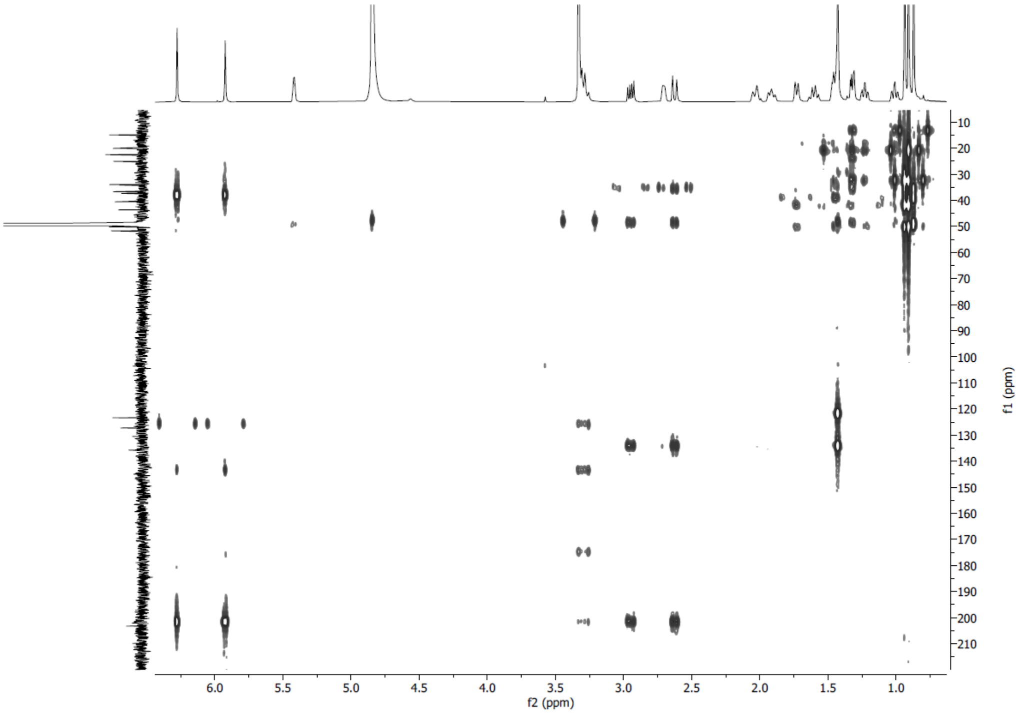


**Figure S5.** HMBC spectrum of compound **1** ($CD_3OD$, 600 MHz).

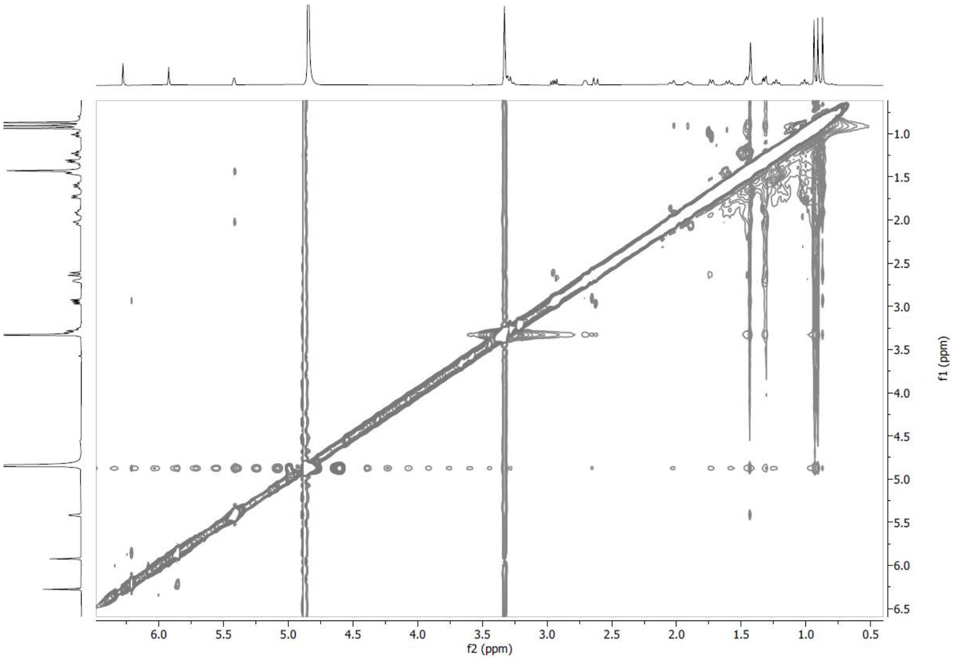


**Figure S6.** NOESY spectrum of compound **1** ($CD_3OD$, 600 MHz)

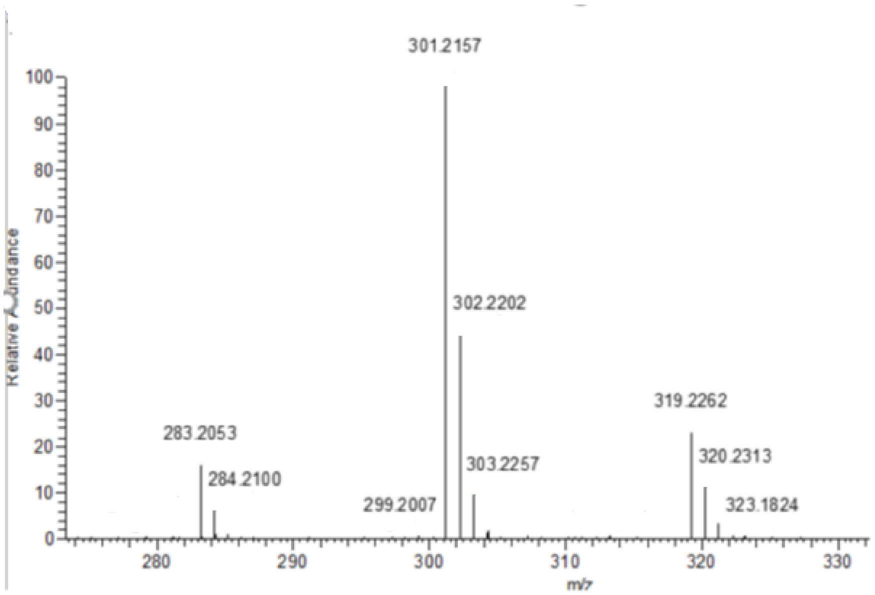


**Figure S7.** HRESIMS of compound **1.**

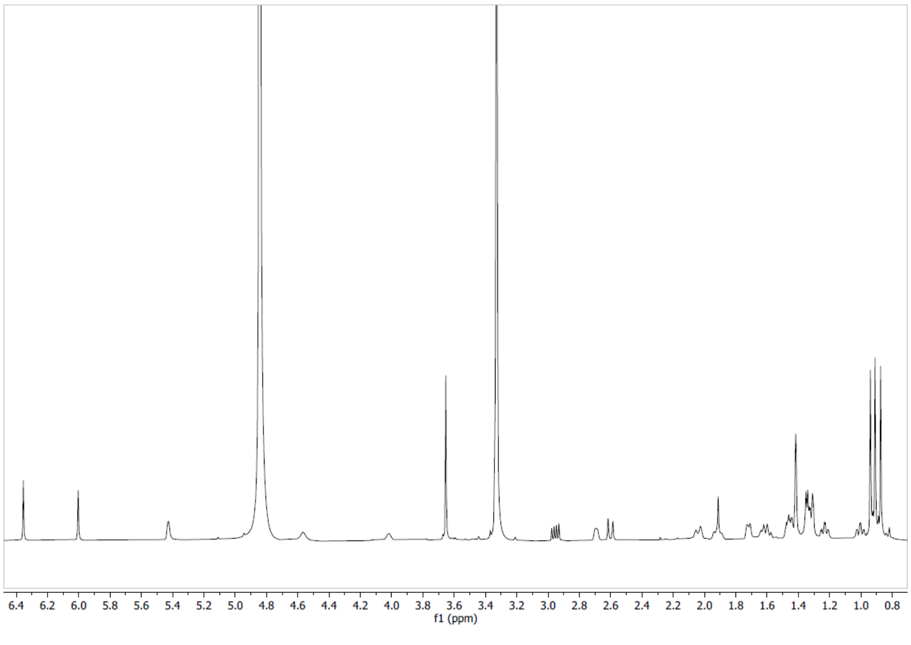


**Figure S8.** $^{1}$H NMR spectrum of compound **2** ($CD_3OD$, 600 MHz).

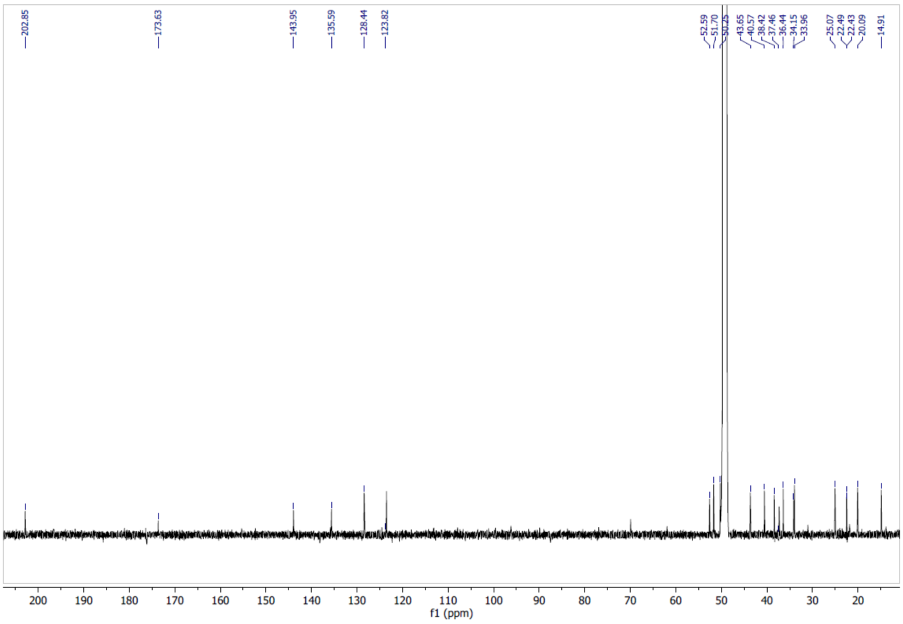


**Figure S9.** $^{13}$C NMR spectrum of compound **2** ($CD_3OD$, 600 MHz).

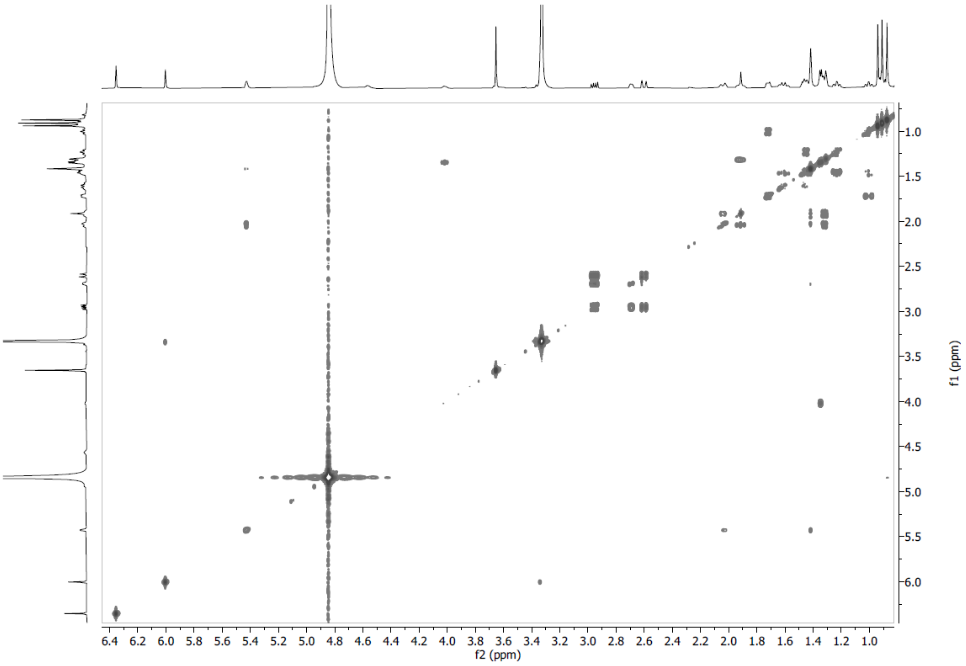


**Figure S10.** COSY spectrum of compound 2 ($CD_3OD$, 600 MHz).

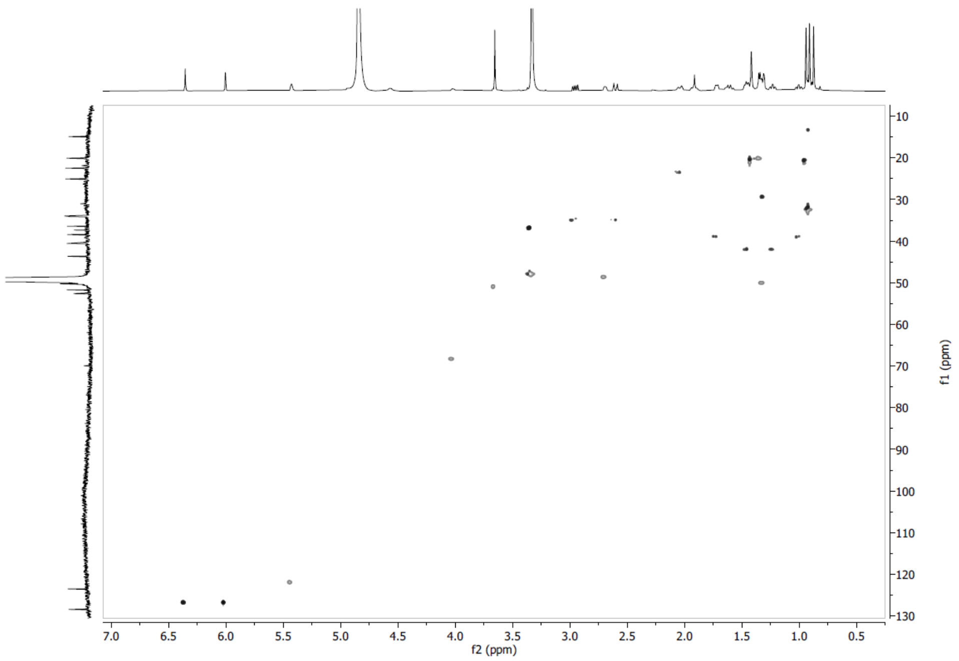


**Figure S11.** HSQC spectrum of compound **2** ($CD_3OD$, 600 MHz).

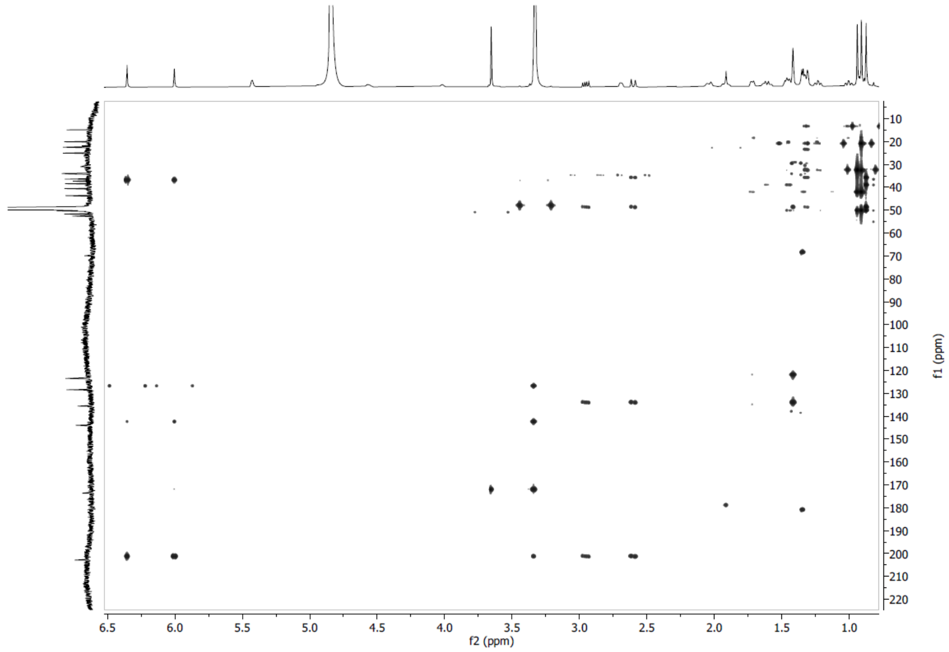


**Figure S12.** HMBC spectrum of compound 2 ($CD_3OD$, 600 MHz).

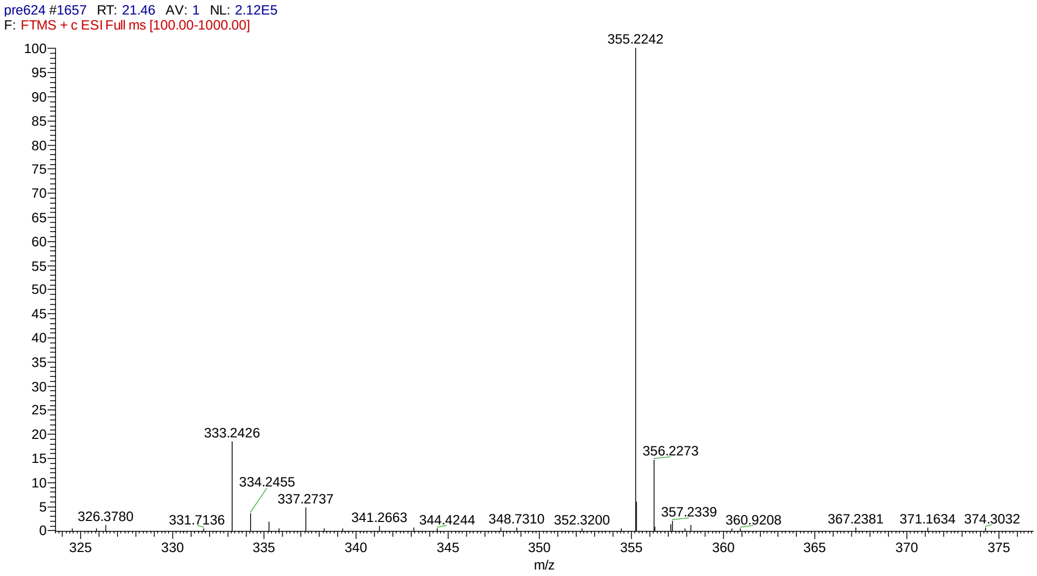


**Figure S13.** HRESIMS of compound **2.**

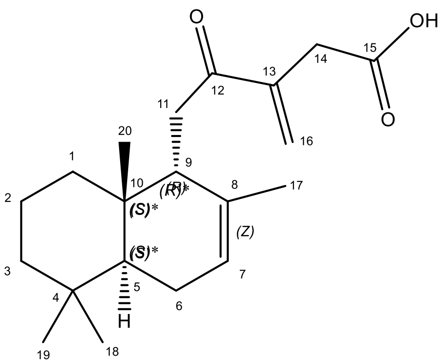


**1a** (5*S**, 9*R**, 10*S**)

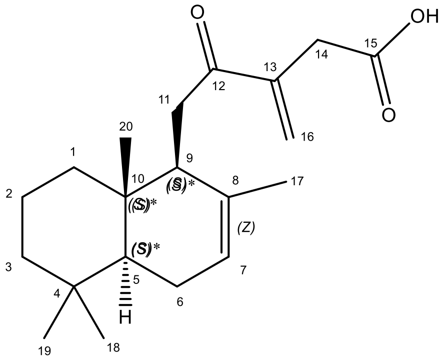


**1b** (5*S**, 9*S**, 10*S**)

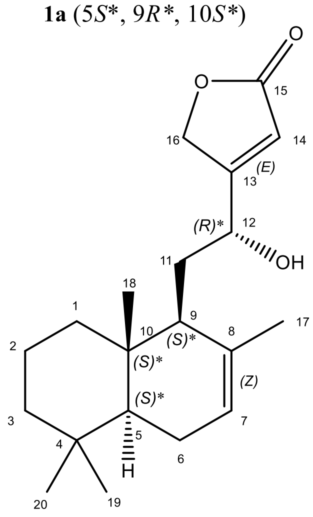


**4a** (5*S**, 9*S**, 10*S**, 12*R**)

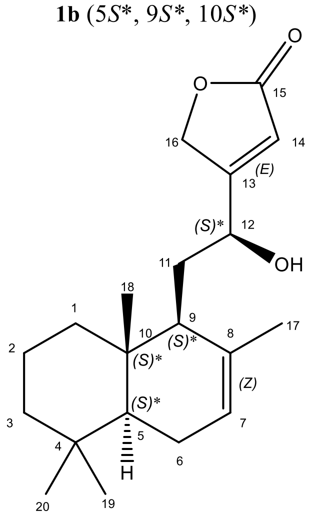


**4b** (5*S**, 9*S**, 10*S**, 12*S**)

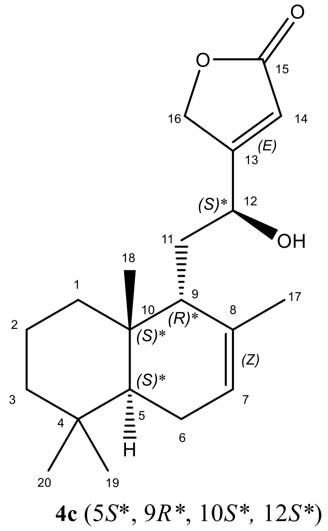


**4c** (5*S**, 9*R**, 10*S**, 12*S**)

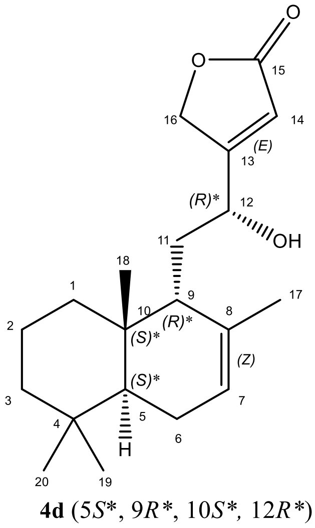


**4d** (5*S**, 9*R**, 10*S**, 12*R**)

**Figure S14.** 2D structures of investigated stereoisomers of **1** and **4**.

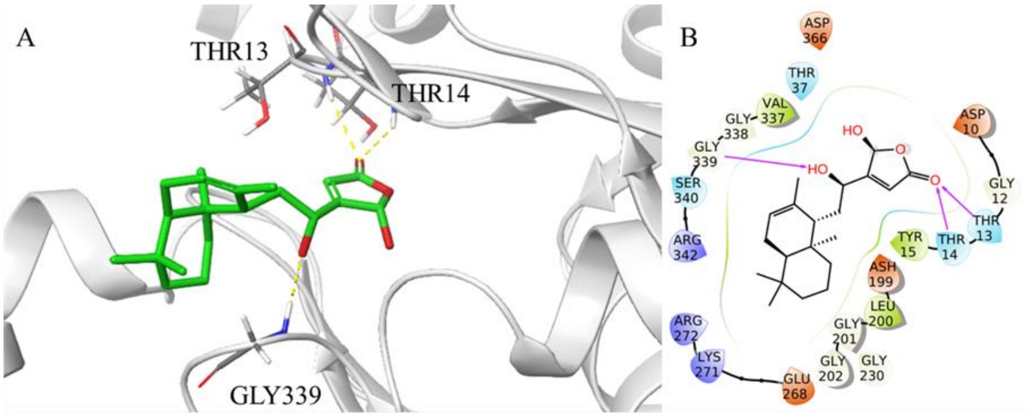


**Figure S15.** Binding pose and interaction of **3** docked to Hsp70 ATP binding site. (**A**) The protein is reported as grey ribbons and residues are colored by atom types; the ligand is reported as green capped sticks; H-bonds are presented as yellow dotted lines. (**B**) The ligand is surrounded by the protein residues represented as follows: the negatively charged residues are indicated in red, polar residues are in cyan, hydrophobic residues are shown in green; H-bonds are depicted as purple arrows.

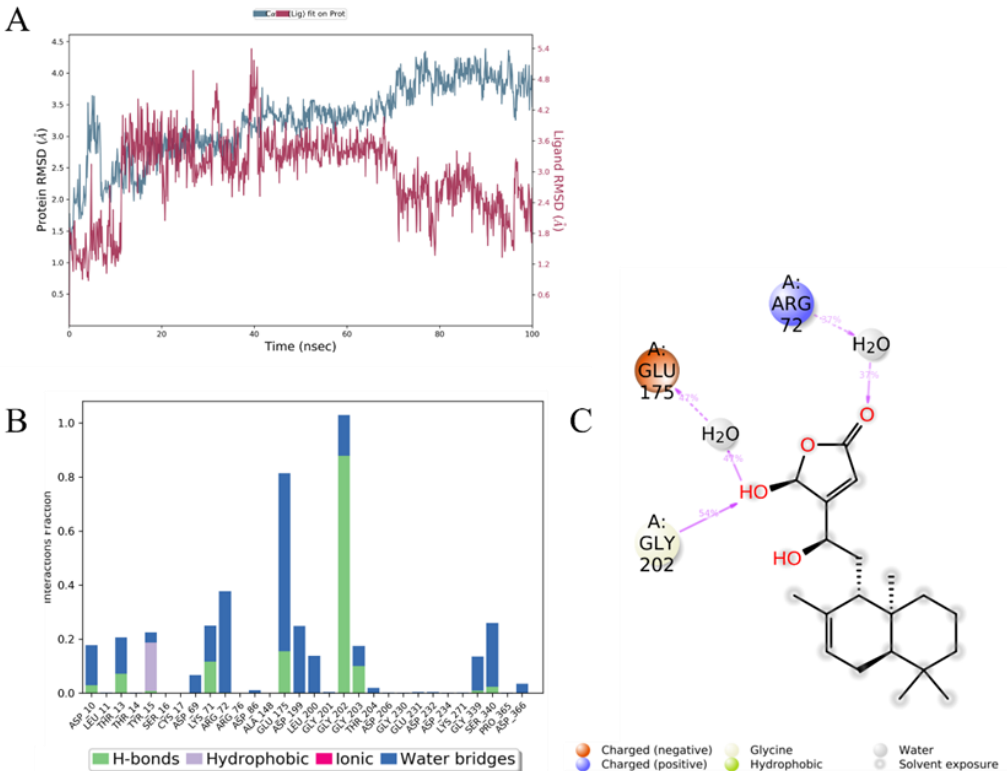


**Figure S16.** Molecular dynamic simulation results. (**A**) Root-mean square deviation (RMSD) plot for **3**/Hsp70 complex along 100 ns molecular dynamics simulation related to C$\alpha$ positions of residues belonging to the protein backbone (blue) and the ligand (purple). (**B**) Protein-ligand interactions (or 'contacts') plot for **3**/Hsp70 complex along 100 ns molecular dynamics simulation. Contacts are categorized into four types: hydrogen bonds, hydrophobic, ionic and water bridges. (**C**) Ligand atom interactions with the protein residues. Interactions that occur more than 30.0% of the simulation time in the selected trajectory (0.00 through 100.00 ns), are shown.

**Table S1.** $^1H$ experimental and calculated NMR chemical shifts for 1**a**-**b**, with [a]|Δδ|($^1H$) and [c]MAE values. Chemical shift data here reported were produced using benzene as reference compound for $sp^2$ hydrogens, and tetramethylsilane (TMS) for $sp^3$ hydrogens.

| $^1H$ | $\delta_{exp}(^1H)$, ppm | $\delta_{calc}(^1H)$, ppm | | \|Δδ\| ($^1H$), ppm | |
|---|---|---|---|---|---|
| **Position** | **exp_1** | **calc_1a** | **calc_2b** | **calc_1a** | **calc_1b** |
| 1 | 1.74 | 1.00 | 1.64 | 0.74 | 0.10 |
| 1 | 1.01 | 0.87 | 0.99 | 0.14 | 0.02 |
| 2 | 1.46 | 1.68 | 1.69 | 0.22 | 0.23 |
| 2 | 1.5 | 1.22 | 1.32 | 0.28 | 0.18 |
| 3 | 1.46 | 1.19 | 1.24 | 0.27 | 0.22 |
| 3 | 1.24 | 1.32 | 1.36 | 0.08 | 0.12 |
| 5 | 1.33 | 1.38 | 1.38 | 0.05 | 0.05 |
| 6 | 1.96 | 2.07 | 2.03 | 0.11 | 0.07 |
| 6 | 2.05 | 2.07 | 2.13 | 0.02 | 0.08 |
| 7 | 5.43 | 5.37 | 5.75 | 0.06 | 0.02 |
| 9 | 2.71 | 2.56 | 2.81 | 0.15 | 0.10 |
| 11 | 2.94 | 3.32 | 2.95 | 0.38 | 0.01 |
| 11 | 2.63 | 2.31 | 2.61 | 0.32 | 0.02 |
| 14 | 3.3 | 3.17 | 3.22 | 0.13 | 0.08 |
| 16 | 5.94 | 6.14 | 6.46 | 0.20 | 0.52 |
| 16 | 6.3 | 6.43 | 6.75 | 0.13 | 0.11 |
| 17 | 1.46 | 1.61 | 1.34 | 0.15 | 0.12 |
| 18 | 0.93 | 0.97 | 0.98 | 0.04 | 0.05 |
| 19 | 0.96 | 0.87 | 0.88 | 0.09 | 0.08 |
| 20 | 0.87 | 1.09 | 0.98 | 0.22 | 0.11 |
| **MAE** | | | | 0.19 | 0.11 |

**Table S2.** $^{13}C$ experimental and calculated NMR chemical shifts for **1a**-**b**, with [a]|Δδ|($^{13}C$) and [b]MAE values. Chemical shift data here reported were produced using benzene as reference compound for $sp^2$ carbons, and tetramethylsilane (TMS) for $sp^3$ carbons.

| $^{13}C$ | $\delta_{exp}(^{13}C)$, ppm | $\delta_{calc}(^{13}C)$, ppm | | \|Δδ\| ($^{13}C$), ppm | |
|---|---|---|---|---|---|
| **Position** | **exp_1** | **calc_1a** | **calc_1b** | **calc_1a** | **calc_1b** |
| 1 | 38.7 | 37.6 | 39.8 | 1.1 | 1.1 |
| 2 | 18.0 | 20.8 | 20.9 | 2.8 | 2.9 |
| 3 | 41.7 | 42.1 | 41.7 | 0.4 | 0.0 |
| 4 | 33.0 | 33.8 | 34.2 | 0.8 | 1.2 |
| 5 | 50.1 | 42.1 | 49.6 | 8.0 | 0.5 |
| 6 | 23.5 | 26.5 | 26.2 | 3.0 | 2.7 |
| 7 | 122.0 | 126.3 | 126.3 | 4.3 | 4.3 |
| 8 | 134.0 | 138.9 | 138.9 | 4.9 | 4.9 |
| 9 | 48.6 | 47.1 | 49.0 | 1.5 | 0.4 |
| 10 | 36.0 | 37.8 | 37.5 | 1.8 | 1.5 |
| 11 | 35.0 | 39.7 | 36.9 | 4.7 | 1.9 |
| 12 | 203.0 | 201.3 | 201.3 | 1.7 | 1.7 |
| 13 | 144.0 | 144.8 | 144.8 | 0.8 | 0.8 |
| 14 | 37.2 | 39.5 | 38.6 | 2.3 | 1.4 |
| 15 | 174.5 | 171.2 | 171.2 | 3.3 | 3.3 |
| 16 | 125.5 | 134.5 | 134.5 | 9.0 | 9.0 |
| 17 | 21.1 | 24.1 | 23.8 | 3.0 | 2.7 |
| 18 | 32.0 | 33.6 | 33.9 | 1.6 | 1.9 |
| 19 | 21.0 | 23.1 | 22.9 | 2.1 | 1.9 |
| 20 | 13.0 | 22.8 | 16.7 | 9.8 | 3.7 |
| **MAE** | | | | 3.3 | 2.4 |

**Table S3.** $^1$H experimental and calculated NMR chemical shifts for **4a**-**d**, with [a]|Δδ|($^1$H) and [c]MAE values. Chemical shift data here reported were produced using benzene as reference compound for sp$^2$ hydrogens, and tetramethylsilane (TMS) for sp$^3$ hydrogens.

| $^1$H | $\delta_{exp}$($^1$H), ppm | $\delta_{calc}$($^1$H), ppm | | | | \|Δδ\| ($^1$H), ppm | | | |
|---|---|---|---|---|---|---|---|---|---|
| **Position** | **exp_4** | **calc_4a** | **calc_4b** | **calc_4c** | **calc_4d** | **calc_4a** | **calc_4b** | **calc_4c** | **calc_4d** |
| 1 | 0.87 | 1.02 | 1.11 | 1.71 | 1.54 | 0.15 | 0.24 | 0.84 | 0.67 |
| 1 | 1.76 | 1.75 | 1.71 | 1.33 | 1.17 | 0.01 | 0.05 | 0.43 | 0.59 |
| 2 | 1.42 | 1.36 | 1.36 | 1.43 | 1.35 | 0.06 | 0.06 | 0.01 | 0.07 |
| 2 | 1.6 | 1.72 | 1.71 | 1.81 | 1.78 | 0.12 | 0.11 | 0.21 | 0.18 |
| 3 | 1.45 | 1.37 | 1.37 | 1.44 | 1.37 | 0.08 | 0.08 | 0.01 | 0.08 |
| 3 | 1.21 | 1.24 | 1.25 | 1.35 | 1.27 | 0.03 | 0.04 | 0.14 | 0.06 |
| 5 | 1.25 | 1.36 | 1.38 | 1.65 | 1.64 | 0.11 | 0.13 | 0.40 | 0.39 |
| 6 | 1.91 | 2.03 | 2.05 | 2.22 | 2.15 | 0.12 | 0.14 | 0.31 | 0.24 |
| 6 | 2.02 | 2.15 | 2.13 | 2.16 | 2.10 | 0.13 | 0.11 | 0.14 | 0.08 |
| 7 | 5.48 | 5.46 | 5.49 | 5.67 | 5.53 | 0.02 | 0.01 | 0.19 | 0.05 |
| 9 | 1.87 | 2.38 | 2.28 | 1.72 | 1.51 | 0.51 | 0.41 | 0.15 | 0.36 |
| 11 | 1.71 | 1.61 | 1.56 | 2.01 | 1.96 | 0.16 | 0.15 | 0.24 | 0.19 |
| 11 | 1.77 | 1.46 | 1.82 | 1.83 | 1.60 | 0.25 | 0.05 | 0.12 | 0.11 |
| 12 | 4.71 | 4.60 | 4.44 | 5.44 | 4.91 | 0.11 | 0.27 | 0.73 | 0.20 |
| 14 | 6.05 | 5.72 | 5.70 | 5.68 | 5.72 | 0.33 | 0.35 | 0.37 | 0.33 |
| 16 | 5.01 | 4.89 | 4.90 | 4.91 | 4.91 | 0.12 | 0.11 | 0.10 | 0.10 |
| 16 | 5.03 | 4.86 | 4.93 | 4.90 | 4.95 | 0.17 | 0.10 | 0.13 | 0.08 |
| 17 | 1.78 | 1.70 | 1.66 | 1.87 | 1.79 | 0.08 | 0.12 | 0.09 | 0.01 |
| 18 | 0.89 | 0.86 | 0.87 | 0.91 | 0.87 | 0.03 | 0.02 | 0.02 | 0.02 |
| 19 | 0.93 | 0.97 | 0.99 | 1.01 | 0.98 | 0.04 | 0.06 | 0.08 | 0.05 |
| 20 | 0.83 | 0.93 | 0.97 | 1.04 | 1.04 | 0.10 | 0.14 | 0.21 | 0.21 |
| **MAE** | | | | | | 0.13 | 0.13 | 0.23 | 0.19 |

**Table S4.** $^{13}C$ experimental and calculated NMR chemical shifts for **4a**-**d**, with [a]|Δδ|($^{13}C$) and [b]MAE values. Chemical shift data here reported were produced using benzene as reference compound for sp$^2$ carbons, and tetramethylsilane (TMS) for sp$^3$ carbons.

| $^{13}C$ | $\delta_{exp}(^{13}C)$, ppm | $\delta_{calc}(^{13}C)$, ppm | | | | \|Δδ\| ($^{13}C$), ppm | | | |
|---|---|---|---|---|---|---|---|---|---|
| **Position** | **exp_4** | **calc_4a** | **calc_4b** | **calc_4c** | **calc_4d** | **calc_4a** | **calc_4b** | **calc_4c** | **calc_4d** |
| 1 | 39.0 | 39.3 | 39.5 | 37.5 | 36.0 | 0.3 | 0.5 | 1.5 | 3.0 |
| 2 | 18.4 | 20.8 | 20.8 | 20.9 | 20.9 | 2.4 | 2.4 | 2.5 | 2.5 |
| 3 | 42.0 | 41.6 | 41.6 | 42.3 | 42.2 | 0.4 | 0.4 | 0.3 | 0.2 |
| 4 | 36.4 | 34.2 | 34.0 | 34.5 | 34.1 | 2.2 | 2.4 | 1.9 | 2.3 |
| 5 | 50.1 | 49.5 | 49.8 | 45.2 | 42.0 | 0.6 | 0.3 | 4.9 | 8.1 |
| 6 | 23.4 | 26.3 | 26.4 | 26.7 | 26.9 | 2.9 | 3.0 | 3.3 | 3.5 |
| 7 | 122.3 | 127.5 | 127.4 | 130.2 | 129.0 | 5.2 | 5.1 | 7.9 | 6.7 |
| 8 | 134.8 | 138.4 | 138.3 | 140.8 | 138.9 | 3.6 | 3.5 | 6.0 | 4.1 |
| 9 | 50.3 | 49.6 | 52.3 | 51.0 | 51.5 | 0.7 | 2.0 | 0.7 | 1.2 |
| 10 | 36.0 | 37.9 | 38.9 | 38.2 | 38.7 | 1.9 | 2.9 | 2.2 | 2.7 |
| 11 | 33.3 | 35.2 | 32.8 | 36.5 | 40.7 | 1.9 | 0.5 | 3.2 | 7.4 |
| 12 | 69.1 | 70.0 | 71.3 | 69.9 | 69.7 | 0.9 | 2.2 | 0.8 | 0.6 |
| 13 | 176.0 | 179.9 | 178.8 | 179.8 | 179.5 | 3.9 | 2.8 | 3.8 | 3.5 |
| 14 | 114.5 | 117.0 | 117.3 | 115.8 | 116.5 | 2.5 | 2.8 | 1.3 | 2.0 |
| 15 | 176.5 | 172.5 | 172.6 | 172.9 | 172.8 | 4.0 | 3.9 | 3.6 | 3.7 |
| 16 | 71.5 | 70.3 | 70.5 | 69.9 | 70.1 | 1.2 | 1.0 | 1.6 | 1.4 |
| 17 | 21.4 | 23.6 | 24.5 | 25.2 | 25.7 | 2.2 | 3.1 | 3.8 | 4.3 |
| 18 | 32.0 | 33.8 | 33.9 | 33.9 | 33.3 | 1.8 | 1.9 | 1.9 | 1.3 |
| 19 | 21.0 | 23.1 | 22.9 | 23.6 | 23.0 | 2.1 | 1.9 | 2.6 | 2.0 |
| 20 | 13.0 | 15.9 | 15.5 | 23.6 | 22.8 | 2.9 | 2.5 | 10.6 | 9.8 |
| **MAE** | | | | | | 2.2 | 2.3 | 3.2 | 3.5 |
